# Dr. Salisbury’s Discovery of the Cause of Intermittent Fever

**Published:** 1866-06

**Authors:** 


### *Dr. Salisbury's Discovery of the Cause of Intermittent Fever.*

IT has come to be almost a matter of course in medical science that any one claiming to have made an important discovery is met at once by a counter-claim, by somebody who has anticipated him in every particular. There is either nothing in it, or it was known before. We ought not to be surprised, therefore, to find that the remarkable announcement of Dr. J. H. Salisbury, which we noticed some weeks since, that he had discovered the hitherto unknown cause of intermittent fever, has called forth a counter-claim. It is from the other side of the ocean, from France, that this claim comes. It is a discovery, it seems, announced so long since as August, 1864, and yet the medical world has been in profound ignorance of the fact until now! A few weeks since our editorial was made the subject of an article in the Union Medicale, and the discovery was laid before the readers of that journal as one of remarkable importance, and sure, if confirmed, to confer great distinction on the discoverer. The article has called out a communication from M. Lemaire, which the Union for March 27th publishes as follows:

"It is with no less satisfaction that we enter a claim of priority concerning Professor Salisbury's discovery of the causative agent of intermittent fever, published in the last number but one. It ought never to be an ungrateful task to avow one's ignorance, to repair an error or omission; it is much more pardonable than bad faith or partiality. We make the *amende* here all the more cheerfully that it has for its object the investigations of a laborious and distinguished confrere, Dr. Lemaire, the author of the Treatise on Phenic Acid, to whom we give place to clear up the history of this question.

"'In 1861, I demonstrated at the Museum of Paris to Professor Gratiolet, Doctors Senechal and Desmarets, assistants, that the gases which are disengaged from matters in an advanced stage of putrefaction always contain, in the vapor accompanying them, either spores or other germinal bodies of microphytes or microzoa. It is sufficient to condense this vapor by cold and to examine it under the microscope to demonstrate this.

"'I published the result of my first researches in the Moniteur Scientifique of Dr. Quesneville in 1862, in the number for the 15th of October. I made use of this discovery, at the time, in that journal, to maintain that the miasms which give origin to marsh fevers, etc., are living organisms. This paper was republished in the first edition of my book on Phenic Acid.

"'In 1864, leaving my laboratory, I went to Sologne with my friend Professor Gratiolet, to repeat my experiments on the miasms which are disengaged from the numerous swamps of that region. We chose those which were reputed the most unhealthy by the inhabitants.

"'We condensed, by means of cold, at the height of a metre [about 39⅓ inches] above the level of the marshes, the vapor which was given off; we examined it by our unassisted senses, by regeants and by the microscope. We observed that at the moment of condensation this liquid contained spherical, ovid and fusiform spores, as well as a great number of pale cells of different dimensions. We found a considerable quantity of very small, semi-transparent bodies, of which I described the forms.

"'You will find the *resume* of these researches, with others not less interesting on the same subject, in the *Comptes rendus* of the Academy of Sciences (sessions of 17th and 29th of August, 1864). I have published an extract from them in the second edition of my book on Phenic Acid, p. 188 and the following pages, and on page 355.

12

" 'I have much to say with regard to the work which you have noticed, but I reserve my remarks for a full memoir on microphytes and microzoa, for which I have been collecting abundant materials for many years.

" 'I have only wished to demonstrate to you that the honor of this discovery does not belong to Professor Salisbury. I hope that the information which I have given you is sufficient to clear up your mind on this point.

" 'The experiments of Dr. Salisbury offer nothing new except the discovery of the microphytes in the expectoration of the sick. It remains now to be seen if other experiments confirm this assertion of the learned professor. I am, &c.,

J. LEMAIRE.'

"The priority of the discovery is thus incontestable. To France returns all the honor of it, for the vegetable origin of these febrigenous spores is but negatively settled. May their pathogenic interpretation be confirmed, and our learned compatriot thus acquire a new claim to the acknowledgments of all the friends of science."

So much for the claim of priority by Dr. Lemaire. In brief it may be stated to be, that in 1861 he demonstrated the presence of spores and other microscopic bodies in the emanations from putrescent matter; that in 1862 he suggested that fever-causing malaria is due to living organisms; that in 1864 he found microscopic forms of various kinds in the condensed vapor given off from an unhealthy marsh.

These observations are certainly extremely interesting and very important; so far as they go, they confirm Dr. Salisbury's observations most fully and satisfactorily. But we contend that they do not transfer the honor of the discovery of the cause of intermittent fever from America to France, inasmuch as they stop short of demonstration. What Dr. Lemaire calls the only novelty in Dr. Salisbury's observations, contains, in our opinion the great point of the discovery. Dr. Lemaire discovers certain bodies in gaseous emanations, and suggests that malarious diseases are caused by living organisms. Dr. Salisbury has done much more than this. He finds the bodies, eliminates certain distinct species, which he finds in different localities, examines the secretions of intermittent fever patients, detects the same bodies there, experiments with the poisonous algoid in a locality far removed from any spot where the disease had ever been known to exist, and actually *creates* in this way the disease in a number of persons! Perfecting his observations, he is able to make out a number of species of these cryptogamous plants, and to discriminate those which give origin to remittent fever from those which produce intermittent. Surely he is entitled to some credit for all this. We would not deny to Dr. Lemaire any honor which his observations deserve. It is very plain that they had not produced much impression in Europe—if for no other reason, that a journalist in the same city announces Dr. Salisbury's discovery as a novelty of unusual importance, being at the time in complete ignorance of his compatriot's labors. At the same time we would remark that it is no novelty for the intermittent fever to be *theoretically* attributed to an organic cause; but so far as we are at present informed, to Dr. Salisbury belongs the honor (if his observations are confirmed by others) of having *demonstrated*, and so made of practical importance, this wonderful discovery.

We would remark, in closing, that the original article in the Union was evidently based upon our editorial on the subject, although no reference to this Journal appears. If the author had read the paper in full in the American Journal of Medical Sciences we are persuaded he would have seen very much more in it to admire and honor than it was possible for us to introduce into our pages.—*Boston Medical and Surgical Journal.*

